# The significance of small noncoding RNAs in the pathogenesis of cardiovascular diseases

**DOI:** 10.1016/j.gendis.2024.101342

**Published:** 2024-05-30

**Authors:** Hemanyun Bai, Fanji Meng, Kangling Ke, Lingyan Fang, Weize Xu, Haitao Huang, Xiao Liang, Weiyan Li, Fengya Zeng, Can Chen

**Affiliations:** a Department of Cardiology, The Second Affiliated Hospital of Guangdong Medical University, Zhanjiang, Guangdong 524002, China; b Guangdong Medical University, Zhanjiang, Guangdong 524002, China

**Keywords:** Biological function, Biomarkers, Cardiovascular diseases, Gene regulation, SncRNA

## Abstract

With the advancement of high-throughput sequencing and bioinformatics, an increasing number of overlooked small noncoding RNAs (sncRNAs) have emerged. These sncRNAs predominantly comprise transfer RNA-derived fragments (tsRNAs), PIWI-interacting RNAs (piRNAs), Ro-associated non-coding RNAs (RNYs or Y-RNAs), small nucleolar RNAs (snoRNAs), and small nuclear RNAs (snRNAs). Each of these RNA types possesses distinct biological properties and plays specific roles in both physiological and pathological processes. The differential expression of sncRNAs substantially affects the occurrence and progression of various systemic diseases. However, their roles in the cardiovascular system remain unclear. Therefore, understanding the functionality and mechanisms of sncRNAs in the cardiovascular system holds promise for identifying novel targets and strategies for the diagnosis, prevention, and treatment of cardiovascular diseases. This review examines the biological characteristics of sncRNAs and their potential roles in cardiovascular diseases.

## Introduction

According to recent data, the mortality rate of incidental diseases has recently increased despite efforts to address various factors such as environmental health and infectious diseases. However, persistently high fatality rates have led to the emergence of cardiovascular diseases (CVDs) as the global primary cause of human mortality.^1^ Currently, cardiovascular incidents not only frequently occur in middle-aged and elderly populations, but also lead to a noticeable upward trend in mortality rates among young individuals, influenced by long-term lifestyle factors such as sleep patterns and dietary habits.^2^ Hence, preventing and controlling CVD occurrence and progression are crucial to reducing associated mortality rates.

Non-coding RNAs (ncRNAs) do not serve as templates for protein encoding and perform diverse essential biological functions within cells. Based on their length, ncRNAs are broadly classified as long-chain non-coding RNAs (>200 nucleotides), including long non-coding RNAs (lncRNAs) and circular RNAs, and short-chain non-coding RNAs (sncRNAs) (<200 nucleotides), including microRNAs (miRNAs), small interfering RNAs (siRNAs), transfer RNA-derived fragments (tsRNAs), Ro-associated non-coding RNAs (RNYs or Y RNA)-derived small RNAs, small nucleolar RNAs (snoRNAs), and small nuclear RNAs (snRNAs).^3^ These ncRNAs play pivotal roles in chromatin modification and the regulation of gene expression, thus influencing the physiological and pathological processes of organisms.

NcRNAs play pivotal roles in CVD progression. Owing to their crucial roles, ncRNAs are potential diagnostic and prognostic markers of CVDs.^4^, ^5^, ^6^ Previous investigations have predominantly focused on elucidating the pathogenic mechanisms of ncRNAs, particularly miRNAs in CVDs. These RNA molecules exert influence via complementary binding to the 3′-untranslated region of messenger RNA (mRNA), which induces its degradation and inhibits target mRNA translation to suppress gene expression. Consequently, miRNAs suppress the expression of genes associated with CVDs, thereby affecting the critical functions of cardiac cells and playing a pivotal role in cardiac health and disease progression.

MiRNAs mediate inflammatory, apoptotic, and autophagic processes,^7^ and they also participate in the proliferation and differentiation of cardiac cells.^8^ Additionally, miRNAs contribute to the remodeling and fibrosis of myocardial structures,^9^ thus impacting the development of various CVDs. These activities have positioned miRNAs as diagnostic and therapeutic biomarkers for cardiac diseases, exerting regulatory control over physiological and pathological processes.^10^

However, sequencing technologies have rapidly advanced, and recent focus has shifted to other sncRNAs and their unique effects on various systemic diseases. Therefore, we have listed the sncRNAs relevant to CVDs outlining their important functions (Table 1). This shows that considering novel sncRNAs as research targets for the treatment and diagnosis of CVDs might represent a novel direction for CVD research.

**Table 1:** The summary of sncRNAs related to cardiovascular diseases.

Related sncRNA	Name	Expression	Target	Gene ID	Function	Cardiovascular diseases	Reference
tsRNA	tsRNA-HC83	UP	MIAT (lncRNA)	440823	Promotes CM survival	MI/R	^11^
	tRF^GlnCTG^	UP	FAS	246097	Elevates proliferation and migration of VSMCs	HTN/AS	^12^
	tRF-Gly-GCC	UP	MHC	3106	Elevates EC proliferation and migration	AS	^13^
	tRF-21-NB8PLML3E	DOWN	–	–	Attenuates cardiomyocyte hypertrophy	HCM	^14^
	tRF-1:30-chrM.Met-CAT	UP	–	–	Boosts VMCS proliferation and migration	AD	^15^
	tRNAVal(CAC)/tRNAGly(GCC)	UP	–	–	Inhibits proliferation, migration, EC tube formation	IS	^16^
	tiRNA-Gln-TTG-001	UP	–	–	–	FM	^17^
	AS-tDR-01269	UP	–	–	–	AF	^18^
	AS-tDR-01363	DOWN	TNFRSF1B, CCL5	7133,6352	–	AF	^18^
	AS-tDR-06049	DOWN	–	–	–	AF	^18^
	tRF-60:76-Val-AAC-1-M5	UP	Tnfrsf10b, Bcl2l1	8795,598	Regulates lipid and AS	MI	^19^
	tRF-5014a	UP	ATG5	9474	Regulates autophagy	DCM	^20^
	5′tiRNA-33-CysACA-1	–	NCOA4	102566861	Reduces CM death	SCM	^21^
piRNA	HAAPIR	UP	Tfec	22797	Reduces CM apoptosis	MI	^22^
	piRNA-6426	DOWN	DNMT3B, SOAT1	1789,6646	Inhibits cardiomyocyte dysfunction	HF	^23^
	has-piR-020009/has-piR-006426	DOWN	–	–	–	HF	^24^
	CHAPIR	UP	Parp10	671535	Accelerates pathological hypertrophy and cardiac remodeling	HCM	^25^
	piRNA-63076	UP	Acadm	24158	Promotes PASMC proliferation	PH	^26^
	DQ593039	UP	–	–	Regulates vascular remodeling	PH	^27^
	DQ614630	UP	–	–	Inhibits EC apoptosis	AS	^28^
	piR-823	UP	HDAC1	3065	Regulates vascular remodeling	AD	^29^
yRNA	EV-YF1	UP	Interleukin-10	3586	Suppresses CM death	MI	^30^
	EV-YF1	UP	CXCL1, TNFR1	2912,7132	Alleviates cardiomyocyte hypertrophy	HCM	^31^
	s-RNY1-5p	UP	CAD	790	Promotes apoptosis and inflammation	AS	^32^
snoRNA	14q32 snoRNAs	UP	Fibrillarin	2091	Regulates vascular remodeling	HF	^33^
	ENSRNOT00000079032.1	DOWN	–	–	Inhibits EC apoptosis	AS	^28^
	SNORD113-6	–	MAP2K1	5604	Regulates cell migration	–	^34^
	ACA35/U94	DOWN	–	–	Affects heart development	ToF	^35^
snRNA	U6atac	–	CA2	760	Regulates cardiac electrophysiology	HF	^36^
	ENSRNOT00000081005.1	UP	–	–	Inhibits EC apoptosis	AS	^28^

Note: Acadm, acyl-coenzyme A dehydrogenase; AD, aortic dissection; AF, atrial fibrillation; AS, atherosclerosis; ATG5, autophagy-related 5; Bcl2l1, BCL2-like 1; CA2, carbonic anhydrase 2; CAD, carbamoyl-phosphate synthetase 2, aspartate transcarbamylase, and dihydroorotase; CCL5, C–C motif chemokine ligand 5; CM, cardiomyocyte; CXCL1, C–X–C motif chemokine ligand 1; DCM, diabetic cardiomyopathy; DNMT3B, DNA methyltransferase 3B; ECs, endothelial cells; FM, fulminant myocarditis; HCM, hypertrophic cardiomyopathy; HDAC1, histone deacetylase 1; HF, heart failure; HTN, hypertension; IS, ischemic stroke; MAP2K1, mitogen-activated protein kinase kinase 1; MHC, major histocompatibility complex; MI, myocardial infarction; MIAT, myocardial infarction-associated transcript; MI/R, myocardial ischemia/reperfusion injury; NCOA4, nuclear receptor coactivator 4; PARP10, poly (ADP-ribose) polymerase family member 10; PASMC, pulmonary arterial smooth muscle cell; PH, pulmonary hypertension; piRNA, PIWI-interacting RNAs; SCM, septic cardiomyopathy; sncRNA, small noncoding RNA; snoRNA, small nucleolar RNA; snRNA, small nuclear RNA; SOAT1, sterol o-acyltransferase 1; Tfec, transcription factor EC; TNFRSF1B, tumor necrosis factor receptor superfamily member 1B; Tnfrsf10b, tumor necrosis factor receptor superfamily 10B; TNFR1, tumor necrosis factor receptor 1; ToF, tetralogy of Fallot; tsRNA, transfer RNA-derived fragment; VSMCs, vascular smooth muscle cells.

## Classification of sncRNAs

### tsRNAs

#### Biogenesis and function of tsRNAs

**Figure 1:**
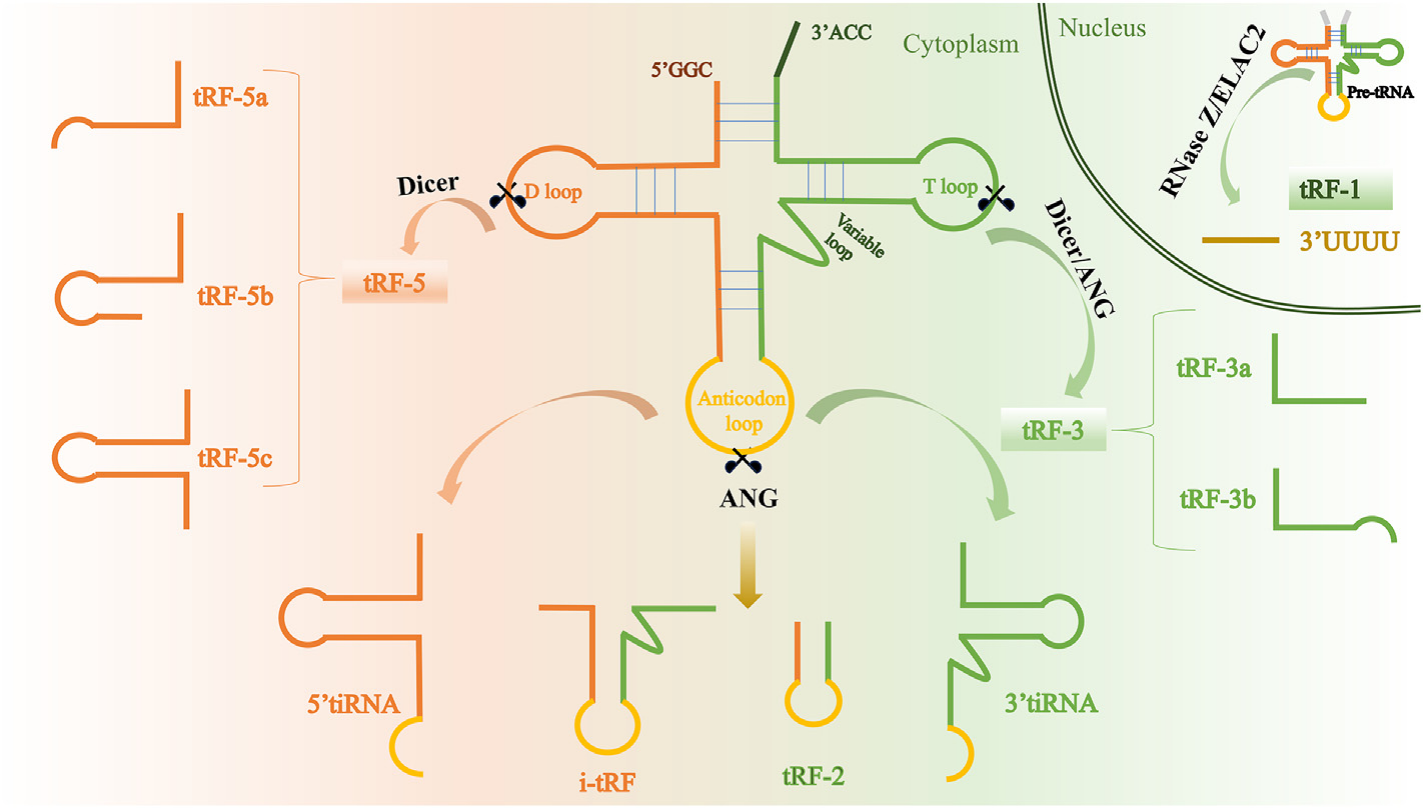
The classification of tsRNAs. tsRNAs include tiRNA and tRF. Under stress, angiogenin cleavage of the tRNA anticodon loop generates tiRNAs that are further categorized as 5′ tiRNA and 3′ tiRNA. Five types of tRF are generated: tRF-1, precursor tRNA produced by RNAse ZL/ELAC2 mediation in the cell nucleus; tRF-3a and tRF-3b originate from T-loop of mature tRNA; tRF-5a, b, and c subtypes are derived from D-loop of tRNA; tRF-2 and i-tRF are generated from anticodon loop of mature tRNA. The orange in the figure represents the pathway generated after D-loop cleavage, the yellow represents the pathway generated after anticodon loop cleavage, and the green represents the pathway generated after T-loop cleavage. ANG, angiogenin; tRNA, transfer RNA; tiRNA, tRNA-derived stress-induced RNAs, tRF, tRNA-derived fragments; tsRNAs, transfer RNA-derived fragments.

Transfer RNA-derived fragments (tsRNAs) are crucial conveyors during translation. They deliver amino acids to ribosomes and play pivotal roles in biological processes. Through specific cleavage by angiogenin, Dicer, RNase Z, or other ribonucleases under stress stimulation at distinct sites, tRNAs generate a class of sncRNAs called tsRNAs^37^^,^^38^ that are typically within the range of 18–40 nt. Based on their cleavage sites and lengths, tsRNAs are broadly categorized as tRNA-derived fragments (tRFs) and tRNA-derived stress-induced RNAs (tiRNAs).^39^ The tRFs typically comprise 14–30 nt and originate from mature or precursor tRNAs. They are usually divided into four subtypes: tRF-5, formed by cleavage between D- and anti-codon loops; tRF-3, generated by T-loop cleavage; 2-tRF (i-tRF), and tRF-1, produced by precursor tRNAs under RNase Z stimulation. In contrast, 30–50 nt tiRNAs primarily emerge under oxidative stress, UV irradiation, hypoxia, starvation, heat shock, or viral infection. Under such conditions, the anti-codon loop undergoes specific enzymatic cleavage, giving rise to 5′-tiRNA of 30–35 nt and 3′-tiRNA of 40–50 nt (Fig. 1).^40^

Previous research has also revealed numerous similarities between tsRNAs and miRNAs. For instance, they share similar lengths and are both generated through cleavage by Dicer. Furthermore, in terms of biogenesis, both can regulate gene expression by binding with argonaute (AGO) proteins, thereby participating in the intricate regulation of physiological and pathological processes.^41^ However, early research on tsRNAs still lacked in-depth investigation, leading many researchers to mistakenly classify tsRNAs as miRNAs. As researchers continued to explore, the differences between the two gradually emerged. Although both tsRNAs and miRNAs bind to AGO proteins, miRNAs typically function by interacting with the 3′-untranslated region of mRNA, thereby inhibiting translation or promoting the degradation of the target genes. In contrast, tsRNAs not only share similar mechanisms, participating in RNAi pathways, but they can also interact with RNA-induced silencing complexes, engaging in the regulation of transcriptional gene silencing post-transcriptionally, thereby suppressing the transcription of certain genes.^42^

TsRNAs can regulate physiological and pathological processes through diverse biological functions. For example, they participate in epigenetic modifications,^43^ modulate the immune response, promote the polarization of M2 macrophages, and are involved in scar formation.^44^ tsRNAs also regulate various signaling pathways, inducing inflammation through the TGF-β signaling pathway and thus affecting skeletal muscle regeneration,^45^ and function in endogenous mitochondrial apoptotic pathways. Under stress conditions, the increased generation of angiogenin competes with cytochrome c for binding, inhibiting the formation of apoptotic bodies and consequently reducing cellular apoptosis.^46^ Similarly, based on their biological characteristics, tsRNAs and miRNAs share similarities in function. miRNAs have also been found to participate in the regulation of various CVDs, including acute myocardial infarction, heart failure, and arrhythmias. Therefore, miRNAs and tsRNAs also exhibit some functional overlap in regulating biological processes such as the cell cycle, apoptosis, and cellular metabolism.^47^

For example, tsRNAs inhibit cellular apoptosis and induce a decline in cellular vitality, leading to apoptotic processes. Under stress, tsRNAs generated by angiogenin (ANG) obstruct ribosomal processing by binding to two ribosomal protein mRNAs. This interruption hampers the processing of small ribosomal protein 28, consequently impairing the maturation of 18S ribosomal RNA (rRNA), and ultimately triggering cellular apoptosis.^48^

Therefore, stress is likely to induce a substantial increase in tsRNAs. According to their distinctive biological nature, tsRNAs simultaneously regulate stress processes through various mechanisms. This phenomenon is prevalent in patients with tumors. For instance, tsRNA expression is elevated in tumor tissues. Overexpressed 5′ tiRNA-His-GTG in colorectal cancer is regulated by hypoxia-inducible factor 1 alpha (HIF1α)/ANG under hypoxic conditions.^49^

In summary, tsRNAs regulate gene expression through various mechanisms that play crucial roles in diverse biological processes and disease development. Consequently, we speculated that differentially expressed tsRNAs are involved in the modulation of CVDs. This is because tsRNA hinders myocardial injury, suppresses cardiomyocyte apoptosis, and promotes cardiomyocyte proliferation through distinct mechanisms. Therefore, it is a potential diagnostic and therapeutic target for CVDs.

#### Roles of tsRNAs in CVDs

The expression of specific tsRNAs is altered in patients with myocardial infarction, heart failure, and atherosclerosis. The apoptosis of myocardial cells plays a pivotal role in CVD development and progression as it results in the loss of functional cardiac tissues, ultimately leading to heart failure. Consequently, methodologies to inhibit myocardial cell apoptosis have become a primary focus of researchers and clinicians. tsRNAs mitigate myocardial damage through various pathways. The RNA-derived small RNA HC83 (tsRNA-HC83) extracted from ginseng alleviates myocardial ischemia-reperfusion injury by selectively binding to and inhibiting the myocardial infarction-associated transcript lncRNA. This reverses the decline in hypoxia-reoxygenation induced by vascular endothelial growth factor A and prevents reactive oxygen species from increasing within cells and mitochondria. This indicates that tsRNA can fully utilize its biological characteristics to cope with stress environments, playing a role like miRNA. By binding to myocardial infarction-associated transcript lncRNA and inhibiting its expression, it can improve heart function and exert a protective effect on the heart.^11^

In fact, the role of tsRNA in CVDs goes far beyond these. The tRFs Gln-CTG and Gly-GCC actively participate in promoting the proliferation and migration of vascular smooth muscle cells in rats and human umbilical vein endothelial cells. These actions play pivotal roles in regulating the occurrence and progression of vascular diseases such as hypertension and atherosclerosis.^12^^,^^13^ Furthermore, tsRNA engages in vascular development, with tRF-1001 modulating endothelial vascular generation through the tRF-1001/METTL3 (methyltransferase-like 3)/RBPJ (recombination signal binding protein for Ig Kappa J region)-MAML1 (mastermind-like protein 1) signaling pathway, which is crucial for tissue repair and regeneration.^50^ In addition to stimulating vascular generation, tsRNAs also up-regulate fragments derived from tRNA-Val-CAC and tRNA-Gly-GCC in endothelial cells that inhibit cell proliferation, migration, and the formation of blood vessels.^16^

TsRNAs play crucial roles in various cardiovascular disorders. The expression of tsRNA substantially differs between persons with and without myocardial hypertrophy. Additionally, tRF-21-NB8PLML3E is down-regulated in patients with myocardial hypertrophy.^14^ The expression profiles of tRFs/tiRNAs specifically vary between persons with and without aortic dissection^15^ and plasma tsRNA expression differs between children who are healthy and those with fulminant myocarditis. The substantially up-regulated expression of tiRNA-Gln-TTG-001 in patients has new significance in the quest for novel therapeutic approaches.^17^ The occurrence and progression of septic cardiomyopathy are associated with tsRNA variations. The expression of distinct tsRNA subtypes derived from tRNA-Cys-GCA cleavage obviously diverged in a comparison of models with septic cardiomyopathy induced by cecal ligation and puncture with a sham group.^21^ A comprehensive comparison of sequenced plasma tsRNAs from patients with idiopathic pulmonary arterial hypertension with healthy persons found that dysregulated tsRNAs closely correlate with pulmonary arterial hypertension, and play extensive roles in the modulation of cellular metabolic processes.^51^ The expression of tsRNAs (notably tRF-5014a) in diabetic cardiomyopathy induced by high glucose is up-regulated through negative modulation of autophagy-related 5 (ATG5) protein and influences cellular vitality and pro-inflammatory factor release.^20^

Further investigation should confirm tsRNAs as evidence of correlations between diseases and drug efficacy. For instance, overabundant tsRNAs are differentially expressed in patients with rheumatic heart disease with and without atrial fibrillation.^18^ Likewise, tsRNAs are differentially expressed between patients with and without systemic vascular resistance (SVR) to sacubitril/valsartan. Within this variation, a specific tsRNA (tRF-60:76-Val-AAC-1-M5) influences the therapeutic heterogeneity of sacubitril/valsartan by targeting Tnfrsf10b (tumor necrosis factor receptor superfamily 10B) and Bcl2l1 (BCL2-like 1) in lipid and atherosclerosis signaling pathways.^19^ These altered expression profiles are detectable in blood, indicating the potential of tsRNAs as noninvasive tools for diagnosing CVDs. This discovery signifies a novel approach to the diagnosis and treatment of CVDs.

In summary, tsRNA can bind to target genes and affect their expression through its physiological mechanism like miRNA, participate in gene regulation, and play corresponding roles, including participating in processes such as myocardial cell apoptosis, proliferation, and migration of vascular smooth muscle cells, and angiogenesis. Due to their different expression levels in CVDs, it suggests that tsRNAs may play an important role in the occurrence and development of diseases, with potential diagnostic value, which can contribute to the early detection and treatment of CVDs. In addition, they may become new targets for addressing CVDs, providing new opportunities for enhancing cardiovascular health and preventing diseases. Therefore, the study of tsRNA is of great significance in the field of cardiovascular medicine, with the potential to improve diagnosis and treatment options.

### piRNAs

#### Biogenesis and function of piRNAs

**Figure 2:**
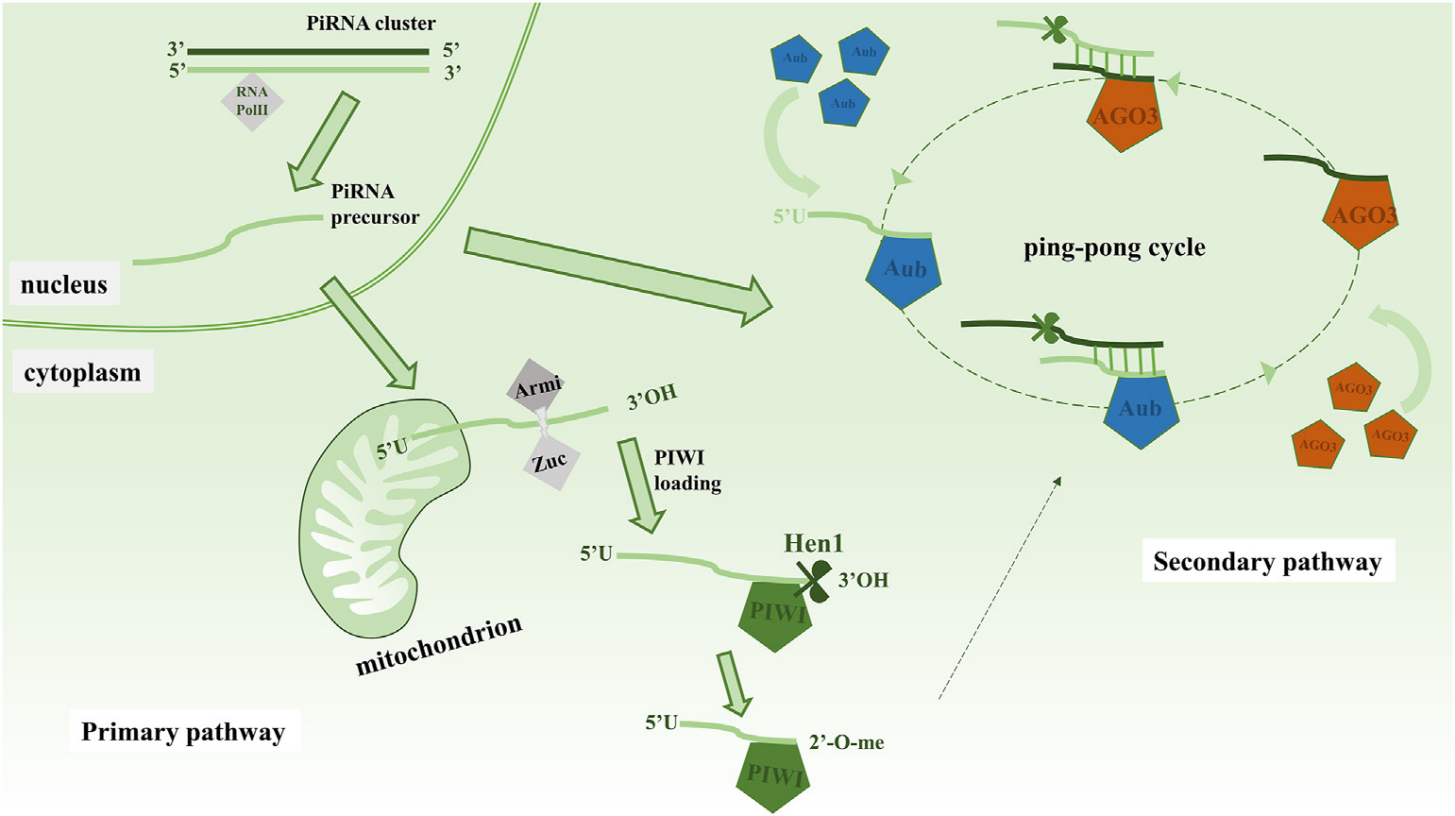
piRNA biogenesis pathways in drosophila. The biogenesis pathways of piRNA involve primary and secondary pathways. The primary pathway entails the transcription of precursor piRNA from piRNA clusters, which is then loaded onto PIWI proteins to form a complex. The secondary pathway, also known as the ping-pong cycle, is initiated by the Aub-piRNA complex, leading to the amplification of the ping-pong mechanism. Subsequently, the mutual cleavage of complementary transcripts from transposons and pre-piRNA results in the generation of a large quantity of piRNA. piRNA, PIWI-interacting RNAs.

The piRNA subtypes of sncRNA comprising 24–31 nt that have a 2′-O-methyl modification site at the 3′ terminus typically manifest a uridine bias (1U-bias) at the 5′ end.^52^ The biological genesis of piRNAs is typically initiated within the cellular nucleus, where RNA polymerase II transforms them from clusters into single-stranded 5′ monophosphorylated piRNA precursors. They are exported from the nucleus to the mitochondria, where they enter primary and ping-pong piRNA pathways (Fig. 2).^53^

**Figure 3:**
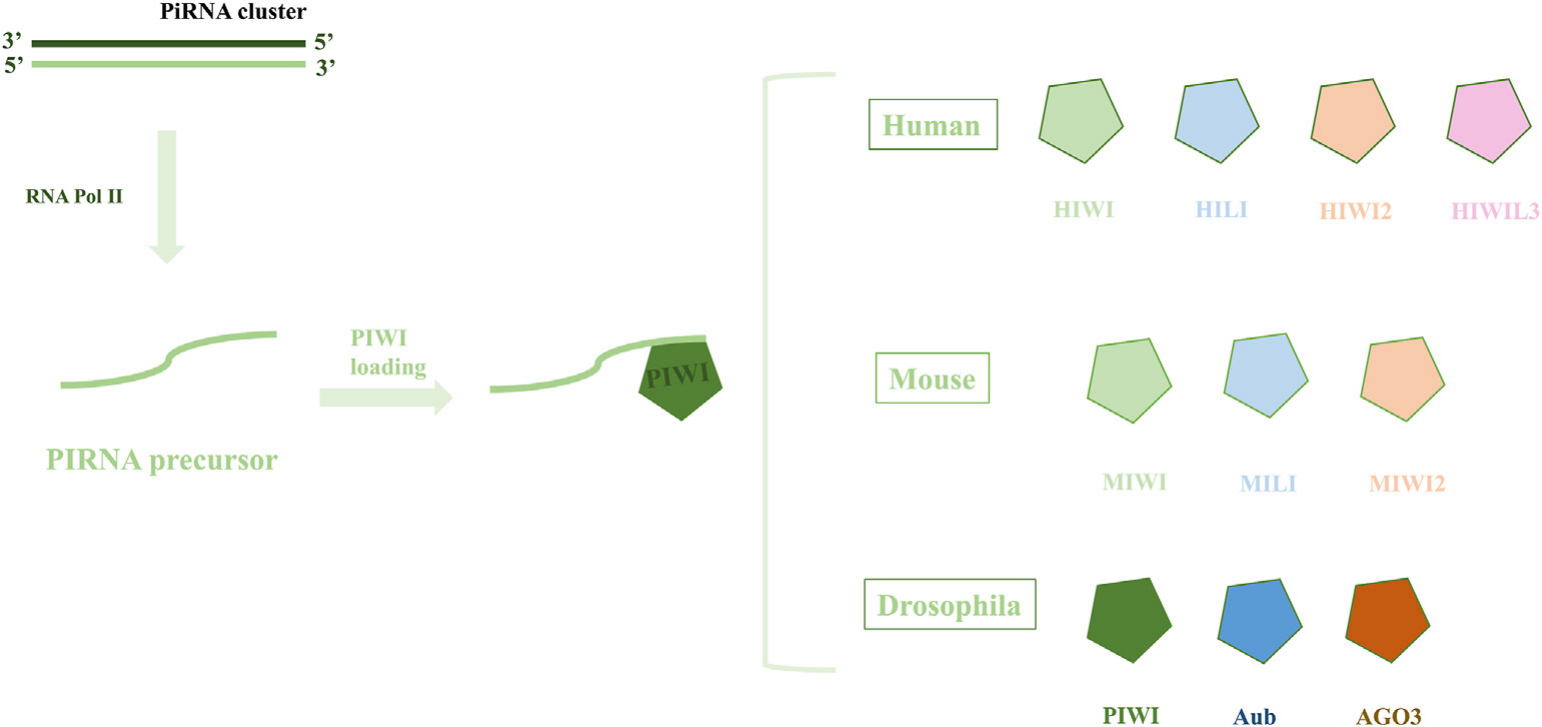
The categorization of PIWI family proteins. piRNA primarily engages in physiological functions by binding with PIWI proteins within reproductive cells, encompassing family proteins in humans, mice, and drosophila. The primary functions of piRNAs include cleaving transposon transcripts to function in post-transcriptional gene silencing, participate in gene regulation during gonadal development, and maintain genomic integrity by suppressing transposable elements (TEs).^55^ piRNA, PIWI-interacting RNAs.

In contrast to the mechanisms involving siRNAs and miRNAs, which bind to the AGO subfamily under the mediation of Dicer, piRNA generation operates independently of Dicer. It employs a distinct mechanism to form a piRNA-induced silencing complex (piRISC) by associating with the Piwi protein. Currently, biosynthesis occurs predominantly in humans, mice, and fruit flies. Variations exist in the PIWI proteins that are associated with piRNAs across different organisms (Fig. 3).^54^

Initially identified predominantly in reproductive cells with increased expression,^56^ accumulating evidence suggests that piRNA expression extends to various other cell types, including the liver,^57^ kidneys,^58^ and brain,^59^ and is notably abundant in cardiac muscle cells.^60^ Through interaction with PIWI proteins, piRNAs play a pivotal role in the developmental processes of organisms, particularly in modulating diverse signaling pathways involved in cellular processes, such as apoptosis, proliferation, and necrosis.^61^

The piRNAs regulate the pathophysiological processes of various systems via different mechanisms and play pivotal roles in the occurrence and progression of various systemic diseases. Up-regulated piRNA expression in tumor cells promotes cell proliferation and induces tumorigenesis,^62^ and thus has the potential to become crucial biomarkers for the diagnosis and treatment of neoplastic conditions.^63^^,^^64^ piRNAs are also associated with immune system disorders, and pirsa-27620 and piR-hsa-27124 expression tend to be high in patients with rheumatoid arthritis, suggesting that these piRNAs could serve as potential diagnostic markers of this disease.^65^ Meanwhile, some studies have found that the abnormal expression of piRNAs is related to the occurrence and development of CVDs, indicating that piRNAs may be involved in the regulation of gene expression and cellular signaling processes. Based on previous research on piRNAs in other systemic diseases, it is fully determined that they have the potential to become an important biomarker for the diagnosis and treatment of heart injury and different types of CVDs, as well as a key target for the diagnosis and treatment of CVDs.^61^^,^^66^ However, compared with tsRNAs and miRNAs, piRNAs have been found to bind to AGO proteins, but their mechanisms of biogenesis are somewhat different. Moreover, research on the role of piRNAs in CVDs is still in its early stages, and the specific mechanism of action still needs further investigation.

#### Roles of piRNAs in CVDs

The expression of piRNAs is increased in the cardiovascular system, suggesting that differential piRNA expression plays a regulatory role in various cardiovascular pathophysiological processes. Increasing evidence supports this suggestion, indicating that piRNA is involved in inducing or resisting apoptosis in myocardial cells, regulating myocardial hypertrophy, resisting inflammation, and promoting angiogenesis. These processes intricately contribute to maintaining homeostasis in the cardiovascular system. Thus, piRNAs could be potential therapeutic avenues for CVDs.

Expression of the heart-apoptosis-associated piRNA (HAAPIR) is abundant in myocardial infarction tissues. Its interaction with N-acetyltransferase 10 (NAT10) increases the N4-acetylcytidine (ac4C) acetylation of transcription factor EC (TFEC) mRNA mediated by NAT10 and consequently elevates TFEC expression. Moreover, TFEC up-regulates transcription of the pro-apoptotic factor B-cell leukemia/lymphoma 2 protein (BCL2)-interacting killer (Bik), which leads to its accumulation. This consequently promotes the progression of the apoptosis pathway, inducing myocardial cell apoptosis, and ultimately causing myocardial damage.^22^ Inhibiting piRNA expression contributes to a degree of repair in myocardial infarction, providing a crucial therapeutic target for addressing myocardial injury.

PiRNAs can also salvage the myocardium by resisting oxidative stress responses and inflammation. Augmenting the expression of piRNA-6426 in myocardial cells induces an increase in DNA methyltransferase 3B (DNMT3B) within the sterol o-acyltransferase 1 (SOAT1) promoter, which consequently suppresses SOAT1 expression and mitigates hypoxia-induced oxidative stress and inflammation in myocardial cells.^23^

We previously identified pivotal protective roles of the p38 protein-mitogen-activated protein kinase (MAPK) and protein kinase B (Akt) pathways in the myocardium after oxidative stress.^67^ Ischemia-reperfusion induction in mice leads to the generation of reactive oxygen species that activate the mammalian target of rapamycin (mTOR) by stimulating the upstream p38 and Akt pathways. Such activation leads to numerous effects, including cell proliferation, resistance to apoptosis, and angiogenesis that maintain normal cardiac function and protect the heart from various injuries.^68^ The proliferation of renal clear-cell carcinoma cells can be stimulated by piRNA-31115 via the phosphoinositide 3-kinase (PI3K)/AKT signaling pathway.^69^ This prompts the question of whether piRNAs similarly induce effects on myocardial cells via this pathway. In fact, the PIWI-piRNA pathway can activate Akt signal transduction in the cardiac system, potentially exerting a protective effect on the heart.^70^ This discovery provided a new theoretical foundation for exploring the roles of piRNAs in the cardiovascular system.

The regulatory role of piRNAs has been determined in experimental models of atherosclerosis induced by oxidized low-density lipoprotein to understand the impact of piRNAs on endothelial cells. Overexpression of the piRNA DQ614630 is associated with an upward trend of apoptosis in endothelial cells, implying a regulatory capacity of piRNAs in governing endothelial cell injury.^28^

Through an intricate association with extracellular vesicles, piRNAs significantly influence intercellular interactions in serum samples from healthy individuals and patients with heart failure. Discrepancies in piRNA expression identified within extracellular vesicles notably included a substantial reduction in the expression of has-piR-020009 and has-piR-006426. This suggests that piRNA expression is altered in tandem with the occurrence of heart failure.^24^

The expression of piRNA differs within extracellular vesicles in serum samples isolated from persons with and without chronic thromboembolic pulmonary hypertension. These piRNA variations are likely to contribute to cardiac and vascular remodeling.^27^ Similarly, piRNA (piRNA-63076) is also differentially expressed under hypoxia; it also modulates the proliferation of pulmonary arterial smooth muscle cells and contributes to the development of pulmonary arterial hypertension by mediating acyl-coenzyme A dehydrogenase (Acadm) methylation.^26^ Furthermore, piR-823 expression is elevated during aortic dissection. The binding and inhibition of histone deacetylase 1 (HDAC1) promotes the proliferation, migration, and phenotypic transformation of vascular smooth muscle cells.^29^

The recently unveiled cardiac-hypertrophy-associated piRNA (CHAPIR) has revealed differential piRNA expression between hypertrophic and healthy murine hearts, thus establishing a regulatory role in mitigating damage caused by cardiac hypertrophy.^25^ Upon forming a complex with PIWIL4, CHAPIR selectively targets METTL3, thereby influencing its RNA methylation activity. This inhibition leads to the up-regulation of poly (ADP-ribose) polymerase family member 10 (Parp10) expression by suppressing the m^6^A modification of Parp10 mRNA, consequently inducing myocardial hypertrophy. Knockdown of CHAPIR attenuates cardiac hypertrophy and restores cardiac function. This study highlights the potential roles of altered piRNA expression profiles in hypertrophic hearts, in terms of regulating gene expression programs linked to cardiac enlargement. In summary, piRNAs have distinctive biological functionalities that offer promising avenues for the deeper comprehension and treatment of CVDs, given their significant impact on the cardiovascular system.

### yRNAs

#### Biogenesis and function of yRNAs

The cytoplasmic RNA yRNAs are typically associated with the Ro ribonucleoprotein complex, with which they form a stable Ro60RNP complex with Ro 60 kD (SS-A/TROVE2) protein.^71^ The interrelation between these entities is intimately connected, with Ro 60 stabilizing yRNA. A reduction in yRNA expression leads to a corresponding decrease in Ro 60 content, substantiating the supportive role of yRNAs in the interaction between Ro 60 and other proteins.^72^ Based on their stem-loop sequences and lengths, yRNAs are classified as the primary types Y1, Y3, Y4, and Y5. Furthermore, yRNAs are ubiquitous and function in various life forms from bacteria to humans.^73^ The expression of yRNAs significantly varies among organs and tissues. Early studies suggested that altered yRNA expression plays significant roles in malignant conditions such as lung cancer,^74^ human papillomavirus infections,^75^ and head and neck squamous cell carcinoma.^76^ Therefore, understanding changes in yRNA expression has crucial implications for the diagnosis and treatment of these diseases.^77^

Initially, yRNAs were identified in the serum of patients with systemic lupus erythematosus. Thereafter, pivotal roles of yRNAs were uncovered in autoimmune diseases, particularly the significant influence of 5′-YsRNA in blood and saliva on Sjögren's syndrome.^78^ With the continuous deepening of research on yRNAs, their roles in CVDs are gradually being discovered. The expression level of yRNAs, especially in the heart and brain, is significantly higher than in other organs, and there is reason to suspect that yRNAs have a significant impact on the cardiovascular system.^32^ For example, when comparing the serum yRNA levels of patients with coronary artery disease with healthy individuals, it was found that yRNA levels were significantly up-regulated in patients with coronary artery disease.

#### Roles of yRNAs in CVDs

Like other sncRNAs, divergent yRNA expression plays pivotal roles in various cellular processes. Although the precise role of yRNA in the cardiovascular system has yet to be thoroughly explored, recent findings suggest its crucial regulatory significance in cardiovascular health and diseases. This is primarily manifested in the modulation of anti-inflammatory and anti-apoptotic responses, myocardial hypertrophy regulation, platelet activation, and the control of coronary artery disease.

Moreover, extracellular vesicles play pivotal roles in intercellular communication and substance exchange, as they house a diverse array of sncRNAs, particularly enriched in yRNAs that participate in various immune-related processes such as inflammation and immune suppression.^79^ This indicates the potential of yRNA derived from extracellular vesicles to serve as a biomarker of immune-related processes.^80^

The yRNA fragment EV-YF1 from extracellular vesicles not only facilitates intercellular communication but also protects the heart by modulating immune responses within myocardial cells.^30^ These extracellular vesicles transport yRNA to macrophages, subsequently inducing a robust protective effect in myocardial cells under oxidative stress by promoting interleukin-10 production. Administering EV-YF1 into the coronary artery during ischemia-reperfusion reduces the area of myocardial infarction, further substantiating the role of yRNAs in resisting cellular apoptosis by regulating inflammatory responses and protecting the heart. Moreover, an association between EV-YF1 and hypertrophic cardiomyopathy is specifically linked to the processes of myocardial cell hypertrophy and fibrosis.^31^ An echocardiographic comparison found that left ventricular wall thickness was significantly alleviated in mice with hypertrophic cardiomyopathy administered with YF1 compared with physiological saline. This suggests a potential effect of YF1 on myocardial hypertrophy. Expression of the inflammation-associated chemokine CXCL1 (C–X–C motif chemokine ligand 1) is reduced by YF1 in myocardial cells *in vitro*. Consequently, YF1 suppresses the expression of pro-inflammatory cytokines, which impedes macrophage infiltration and thus protects the heart.

Platelet equilibrium is a pivotal determinant of the genesis of CVDs. Platelets play decisive roles in hemostasis and thrombus formation and harbor a wealth of sncRNAs, including yRNAs. These yRNAs, together with other ncRNAs, collectively participate in modulating platelet activation and aggregation. Consequently, alterations in yRNA are likely to affect the progression of CVDs such as coronary artery disease and atherosclerosis.^81^

Furthermore, yRNAs are also potential regulatory factors in atherosclerosis,^82^ because they play crucial roles in its detection as well as related processes through alternative pathways. Numerous protein-binding sites in yRNA are pivotal to regulating the developmental course of atherosclerosis because yRNA can inhibit lipid accumulation and foam cell formation, and concurrently participate in regulating inflammation and apoptosis. Activation of the nuclear factor kappa B (NF-κB) and caspase 3 principal apoptosis signaling pathways in these processes under the influence of YRNA-Ro60 complexes leads to inflammatory damage and cell death. This cascade ultimately results in endothelial impairment and the onset of atherosclerosis.^83^

Although a more profound investigation into the mechanistic role of yRNA in CVDs is warranted, the present findings suggest that yRNAs play diverse roles in the development and progression of CVDs. Further exploration is imperative to comprehensively understand how yRNAs contribute to CVDs and whether they could serve as diagnostic biomarkers of CVDs and develop novel intervention methods.

### snoRNAs

#### Biogenesis and function of snoRNAs

The snoRNAs residing within the nucleolus of eukaryotic organisms primarily function as rRNAs and are classified as box C/D and box H/ACA types. Box C/D snoRNAs are characterized by the closed-loop structure formed by box C (RUGAUGA) and box D (CUGA), and include structures similar to, but not conservatively structured, boxes C′ and D′. Conversely, box H/ACA snoRNAs consist of two stem-loops, each featuring a box H (ANANNA) sequence and a 3′ tail-end ACA sequence.^84^ Within the snoRNA subfamily, *small Cajal body-specific* RNAs (scaRNAs) are located in the cytoplasm and are primarily tasked with guiding RNA polymerase II-specific spliceosome snRNAs post-transcriptional modifications. They also play roles in mediating modifications such as 2′-O-ribose methylation and pseudo uridylation in snRNA and rRNA.^85^ snoRNAs are associated with core proteins; box C/D snoRNAs typically bind to fibrillarin, Nop56, Nop58p, and Snu13, whereas box H/ACA snoRNAs associate with Gar1, Cbf5, L7Ae, and Nop10. scaRNAs comprise a distinct subclass of snoRNAs, forming stable snoRNA-ribonucleoprotein complexes by binding to the C/D and H/ACA box RNA proteins, commonly referred to as snoRNPs.^86^

These unique physiological mechanisms enable snoRNAs to participate in various physiological and pathological processes. Removing the ribosomal protein L13a (Rpl13a) snoRNA results in increased insulin secretion, thus stimulating pancreatic effects and influencing systemic glucose homeostasis under metabolic stress.^87^ The novel snoRNA jouvence induces cellular reprogramming toward the stem cell direction, thus promoting cell proliferation and providing a new avenue for cancer treatment.^88^

Beyond their impact on physiological functions, dysregulated snoRNAs play crucial roles in the occurrence and progression of pathological conditions. Changes in snoRNA expression can significantly affect multi-system diseases. For instance, high-throughput sequencing has revealed a notable decrease in snoRNA expression in peripheral T-cell lymphomas. Along with this decrease, the specific snoRNA HBII-239 is significantly increased in diseases such as peripheral T-cell lymphoma not otherwise specified and angioimmunoblastic T-cell lymphoma with a favorable prognosis. This suggests that distinct snoRNAs could serve as potential biomarkers in these lymphomas, with varying diagnostic and prognostic significance for different cell types.^89^ snoRNAs have also been implicated in the regulation of cerebrovascular diseases. Comparisons of sequences have discovered that SNORD115-32, SNORD114-22, and SNORD113-3 are significantly decreased in cerebral cavernous malformations compared with healthy brain tissues. This discovery holds promise for the diagnosis and treatment of cerebral cavernous malformations.^90^ It is not difficult to find through the study of a large number of snoRNAs on other systemic diseases that snoRNAs mainly rely on their role in cellular ribosome biosynthesis, and can also have potential effects on different diseases through their dysregulation. Therefore, it is speculated that snoRNAs may affect the expression and function of genes related to the cardiovascular system by regulating RNA modification and expression levels. However, research on the role of this aspect in the cardiovascular system is still limited, and further research is needed to elucidate the specific mechanism of action of snoRNAs in the cardiovascular system and its role in CVDs.

#### Roles of snoRNAs in CVDs

Although research into snoRNAs in the cardiovascular system remains relatively limited, mounting evidence suggests that their crucial roles are primarily achieved through regulating modifications and processing rRNAs and tRNAs. A recent gene screening of thousands of elderly individuals in the PRavastatin in the Elderly at Risk (PROSPER) study revealed that 14q32 snoRNA expression might independently play a role in CVDs associated with the great saphenous vein and coronary arteries. Subsequent validation using tissue samples further confirmed this notion, emphasizing a close connection between changes in 14q32 snoRNA and vascular remodeling, as well as CVDs.^33^

These mechanisms were assessed by overexpressing or knocking down AF357425/SNORD113-6 snoRNA in fibroblasts. The results confirmed that AF357425/SNORD113-6 snoRNA is involved in targeted processes such as pre-mRNA processing, 2′-O-ribose methylation, and maintaining the stability of a wide range of mRNAs. Inhibiting AF357425/SNORD113-6 snoRNA reduced the methylation of integrin signaling pathway mRNA, leading to decreased mRNA expression, increased degradation, and reduced protein levels, thus altering the functionality of arterial fibroblasts, impacting cardiovascular remodeling, and participating in the progression of CVDs.^91^

Vascular remodeling regulated by snoRNAs is associated with tRNA-derived fragments. Under conditions of ischemia and hypoxia, AF357425/SNORD113-6 snoRNA can selectively act on tRNA, guide tRNA methylation, and reduce tRNA-Leu-TAA cleavage into tRFLeu 47–64, thus participating in the regulation of vascular remodeling.^34^

SnoRNAs also affect endothelial cells. Expression of the snoRNA ENSRNOT00000079032.1 was decreased in an atherosclerosis model induced by oxidized low-density lipoprotein. Overexpressed snoRNA inhibits endothelial cell apoptosis, thus preventing atherosclerosis.^28^ Moreover, snoRNAs are associated with CVDs and the risk of congenital heart disease, stroke, and obesity.^92^^,^^93^ The significant down-regulation of several snoRNAs in myocardial tissues of children with tetralogy of Fallot suggests a close connection with the regulation of heart development.^35^^,^^94^

### snRNAs

#### Biogenesis and function of snRNAs

SnRNAs are a category of small ribonucleic acids ranging from 100 to 300 nt in length. They are primarily categorized as major spliceosomes U1, U2, U4, U5, and U6, and minor spliceosomes, including U5, U11, U12, U4atac, and U6atac. snRNAs in eukaryotic nuclei interact with over 100 proteins to form spliceosomes that participate in splicing RNA by removing introns from pre-mRNAs and transforming them into mature mRNA.^95^ Spliceosomes play crucial roles in the normal development and metabolism of plants^96^ and animals and significantly impact the maintenance of physiological balance in organisms. Reductions or mutations in many snRNAs might trigger various diseases and disrupt the physiological balance.

The loss of snRNA 65 K leads to increased apoptosis of epithelial cells in the small intestine and decreased cell proliferation, collectively disrupting the epithelial tissue structure of the small intestine, and consequently affecting absorption and digestive functions.^97^ Knockdown of snRNA 7SK controls human immunodeficiency virus infection-specific transcription by inhibiting CDK9/cyclin T1 kinase activity, which promotes human immunodeficiency virus occurrence.^98^ A decrease in snRNA U4atac is crucial for human growth, development, and postnatal survival.^99^

All the above further underscores the importance of snRNAs in the regulation of gene expression, highlighting their crucial role in maintaining normal cellular functions and development due to their unique biological characteristics.

#### Roles of snRNAs in CVDs

The impact of snRNAs on the cardiovascular system has not been extensively investigated, but they might play essential roles in various processes affecting cardiovascular health. For instance, significantly increased expression of the snRNA ENSRNOT00000081005.1 inhibits endothelial cell apoptosis in atherosclerosis.^28^ Moreover, knocking down the snRNA U6atac in ventricular myocytes for 24 h significantly increases the amount of apoptosis. This impedes small intron splicing in the Na^+^ channel Scn5a (encoding Nav1.5) and the Ca^2+^ channel Cacna1c (encoding Cav1.2), consequently affecting protein levels of the Nav1.5 and Cav1.2 channels and influencing myocardial electrophysiology. A high proportion of myocardial infarction in the heart substantially impacted these results. This emphasized their significant role in maintaining myocardial cell functionality by regulating the expression of splicing snRNAs.^36^ Although snRNAs might function in gene expression regulation and RNA processing, their precise roles and associated mechanisms in the cardiovascular system require further in-depth investigation. Dysregulated snRNA-mediated processes might cause defects at the molecular and cellular levels, thus negatively affecting cardiovascular health and potentially contributing to the development of CVDs. Therefore, a more comprehensive investigation is urgently required to fully understand the specific mechanisms and significance of snRNAs in cardiovascular health and CVDs.

## Conclusion

Although little is known about the roles and effects of sncRNAs in CVDs, undeniable evidence indicates that they play essential roles in the regulation of gene expression and various biological processes in the cardiovascular system.

Compared with other reviews, we have chosen to explore the potential roles of tsRNAs, piRNAs, yRNAs, snoRNAs, and snRNAs in the cardiovascular system because their research depth is not as extensive as miRNAs and siRNAs. With the rapid development of high-throughput sequencing technology, the important role of these little-known sncRNAs in tumors or immune system diseases has gradually emerged. However, research on sncRNAs in CVDs is still limited. Therefore, we provide an overview and summary of these research articles, and group them according to the types of sncRNAs, introducing the biogenesis and molecular mechanisms of different sncRNAs. In addition, we summarized the important effects of various sncRNA dysregulation on different CVDs through limited research articles and explored their functional regulatory mechanisms in the cardiovascular system based on their unique biological characteristics and potential effects on CVDs. Different types of sncRNAs balance physiological and pathological processes through distinct mechanisms, which is the core of our investigation. We hope to bring new perspectives to readers in the treatment and prevention of CVDs.

Although some sncRNAs are still in the early stages of research, as time progresses, more evidence supports these sncRNAs have become increasingly recognized for their critical participation in regulating gene expression and various biological processes in the cardiovascular system and their potential as diagnostic markers and therapeutic targets for CVDs. However, confirmation of the complex interactions between sncRNAs and cardiovascular health/CVDs awaits more comprehensive research.

Despite the growing body of information regarding the impact of sncRNAs on the cardiovascular system, understanding of CVDs remains limited, and many unknown areas require further exploration.

## Funding

This work was supported by the Zhanjiang Science and Technology Plan Project (No. 2022A01143, 2023E0005, 2022A01149, 2021A05094), Discipline Construction Project of Guangdong Medical University (No. GDMXK2021001), Research Project of Guangdong Provincial Bureau of Traditional Chinese Medicine (No. 20232213), and Postdoctoral Research Project of the Second Affiliated Hospital of Guangdong Medical University (No. 22H01) (all China).

## Author contributions

Hemanyun Bai: writing of the original draft; Fanji Meng: conceptualization; Kangling Ke and Lingyan Fang: investigation and methodology; Weize Xua and Haitao Huang: formal analysis and data curation; Xiao Liang, Weiyan Li, and Fengya Zeng: review, editing, and visualization; Can Chen: supervision and project administration.

## Conflict of interests

The authors declared no conflict of interests.
